# Response of reindeer mating time to climatic variability

**DOI:** 10.1186/s12898-020-00312-8

**Published:** 2020-07-29

**Authors:** Amélie Paoli, Robert B. Weladji, Øystein Holand, Jouko Kumpula

**Affiliations:** 1 Department of Biology, Concordia University, 7141 Sherbrooke Street West, Montreal, QC H4B 1R6 Canada; 2 Department of Animal and Aquacultural Sciences, Norwegian University of Life Sciences, P.O. Box 5003, 1432 Ås, Norway; 3 Natural Resources Institute of Finland (Luke), Reindeer Research Station, 99910 Kaamanen, Finland

**Keywords:** Breeding time, Weather conditions, Climatic variation, Plasticity, *Rangifer tarandus*, Ungulates

## Abstract

**Background:**

The breeding time of many species has changed over the past 2–3 decades in response to climate change. Yet it is a key reproductive trait that affects individual's parturition time and reproductive success, and thereby population dynamics. In order to predict how climate change will affect species’ viability, it is crucial to understand how species base their reproductive efforts on environmental cues.

**Results:**

By using long-term datasets of mating behaviours and copulation dates recorded since 1996 on a semi-domesticated reindeer population, we showed that mating time occurred earlier in response to weather conditions at different key periods in their annual breeding cycle. The mating time occurred earlier following a reducing snow cover in early spring, colder minimum temperatures in the last 2 weeks of July and less precipitation in August-September.

**Conclusions:**

The mediated effect of a reduced snow cover in early spring on improving individuals’ pre-rut body weight through a better availability of late winter food and reduced costs of locomotion on snow would explain that mating time has occurred earlier overtime. A lower level of insect harassment caused by colder maximum temperatures in July might have caused an advance in mating time. Less precipitation in August-September also caused the mating time to occur earlier, although the direct effects of the last two weather variables were not mediated through the pre-rut body weight of individuals. As such, the causal effects of weather conditions on seasonal timing of animals are still unclear and other mechanisms than just body weight might be involved (e.g. socio-biological factors). The plastic response of reindeer mating time to climatic variability, despite supplemental feeding occurring in late April, demonstrated that environmental factors may have a greater influence on reproductive outputs than previously thought in reindeer.

## Background

Breeding time in animals is a strong determinant of offspring viability and reproductive success (birds: [1], fish: [2], mammals: [3] and therefore a key component of population dynamics. Accordingly, a mismatch between species’ timing of reproduction and its environment could have major consequences on offspring production [4] and could compromise the species’ viability. In most seasonally breeding mammals, the annual cycle of daily photoperiod has long been identified as the determinant factor of seasonal breeding, while ambient temperature, nutrition state and behaviour exert a modulator effect [5, 6]. The mating season of ungulates is thus regulated by weather conditions both directly (i.e. as proximate factors) through influencing rut and estrus, and indirectly (as ultimate factors) through survival of the young, both by reducing predation risk [7] and by coinciding with vegetation quality or availability [8]. Indeed, for animals living in seasonal environments, the breeding season is ultimately constrained by a genetic control where mating is precisely timed so that parturition would coincide with long-term patterns of climate as a way to offer a hospitable environment when rearing the young [9, 10], balancing adequately the population’s recruitment rate with the adults' probability of survival to the next breeding season [11]. Seasonal breeders might therefore be more sensitive to large changes in climate. Considering reindeer (*Rangifer tarandus* for example, rutting begins in mid-September and last about one month; gestation lasts for about 7 months over the winter, after which females give birth the following spring. Calving is highly synchronous [12] as within a 10 day period, as much as 80–90% of the calves are born and the calving is completed within 4–5 weeks [13, 14]. Offspring born too early would thus be nursed with a low quality milk produced by mothers that are in negative energy balance due to a low quality vegetation (i.e. low protein content; [8]. Similarly if born too late, young are observed to not be able to use summer green flush up as effectively as early born calves and might therefore lack time to grow and develop sufficiently to overcome winter severity [8]. Small and late born calves were then shown to be more prone to (1) insect harassment and summer heat [15] and (2) predation by bears, golden eagles and other predators [16]. Consequently, individuals born outside the optimal period for births ultimately had lower probabilities to survive [8], jeopardizing their survival and growth, as well as the survival and reproductive success of their mothers [17]. In stochastic environments, a plastic response of mating time to environmental change would thus allow species to optimize their recruitment rate under variable climatic conditions.

The timing of reproduction of many taxa has changed over the past two to three decades in response to climate change (bird: [18], amphibian: [19], fish: [20], mammal: [21, 22], marine species: review by [23]. Such observed responses to climate change, however, appeared to be insufficient to track a rapidly changing environment and has led to reduced offspring viability and reproductive success [4]. The mechanisms underlying such phenological changes are still poorly understood. To understand how climate change will affect species’ viability, it is imperative to understand the link between a species’ reproductive strategies and its environment and to understand how the reproductive traits are directly or indirectly affected by climatic changes. In ungulates and long-lived mammals, however, there are several challenges. First, the long overwinter gestation period of those species may render difficult to find the climatic drivers determining the timing of reproduction, because there might be a substantial time lag between those climatic drivers and the point at which reproduction occurs. Second, a certain climatic driver (e.g. temperature) might induce a plastic response in the timing of reproduction but in opposite directions, depending on the time of the year considered. For instance, warmer temperatures in spring result in an increase of vegetation productivity and lengthened growing seasons, which benefits the reproduction of *Rangifer* [24]. On the other hand, warmer temperatures in summer have increased the level of insect harassment and decreased the body condition of reindeer [25]. A first step to understand the mechanisms behind phenological changes is therefore to identify the critical time windows during which the climatic drivers affect the timing of reproduction [26].

A second step to understand the mechanisms behind phenological changes is to decouple the direct and indirect effects that the weather might have on reproductive traits. Capital breeders such as reindeer rely on body reserves to finance reproduction [6] so they could be affected both directly and indirectly by weather conditions: directly by energetic demands (e.g. thermoregulation [27], locomotion on snow [28] and indirectly through plant productivity that they need to build up their endogenous reserves [24, 29, 30]. Body weight of adults is a good metric to take into consideration effects of both animals’ energy requirements and feeding strategies. Reindeer mating was previously found to be influenced by females’ [31, 32] and males’ body weight [33]. In our study, the indirect effects of weather on mating time will therefore be examined through the pre-rut body weight of individuals (measured in September for both males and females). The Arctic surface air temperatures have warmed at twice the global rate [34, 35]. *Rangifer* are one of the two only ungulate species to have established in the unpredictable and austere Arctic environment and was shown to complete the mating season within 4–5 weeks [14]. Therefore, reindeer is an ideal candidate to answer our study question aiming at identifying the critical periods of the year during which climatic drivers affect mating time. This will be achieved by examining the associations between weather, population variables and mating time and using two long-term datasets, one of males’ mating behaviours and the other of copulation dates, recorded since 1996 on a semi-domesticated reindeer population in Finnish Lapland.

**Fig. 1:**
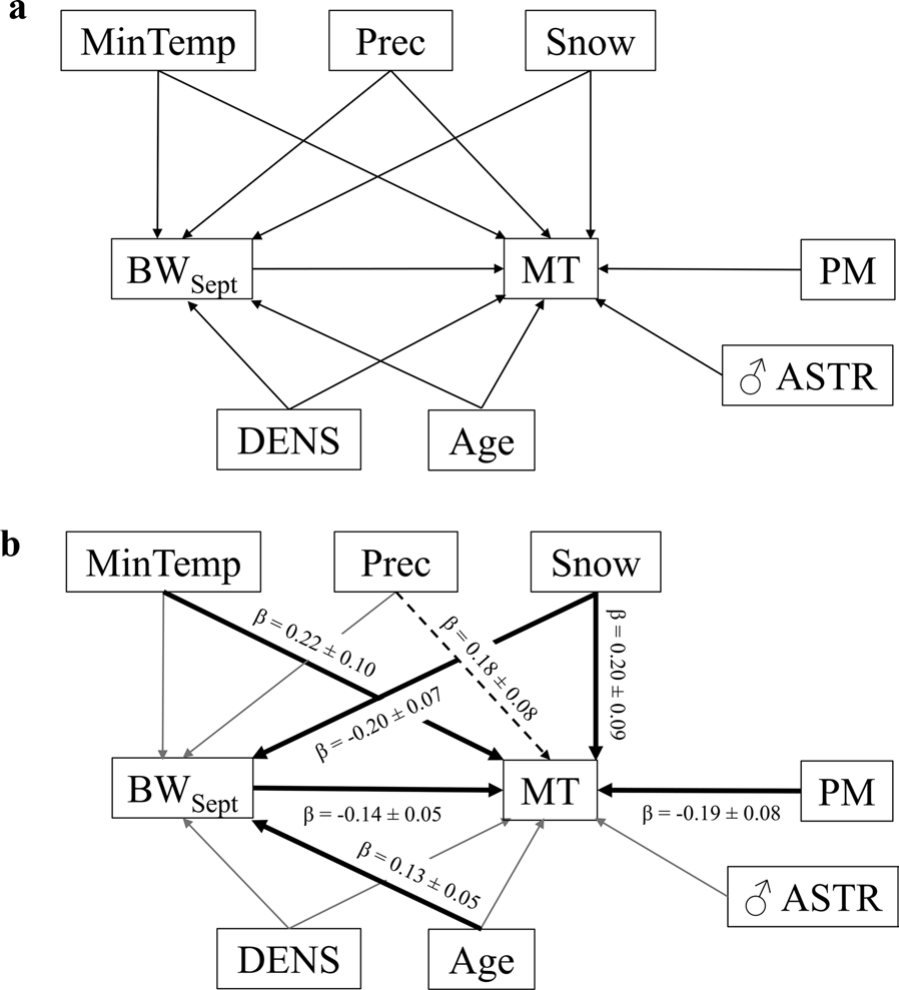
Hypothesized path model for how mating time (‘MT’) of reindeer is affected directly and indirectly by climatic variability from 1996 to 2013 in the Kutuharju herd, northern Finland. The definitions and time windows of the weather variables (‘MinTemp’, ‘Prec’, ‘Snow’) are provided in the *Methods* section, as well as the explanation of (**a**) the hypothesized paths. ‘BW_Sept_’ represents the pre-rut body weight of males and females (measured in September), ‘DENS’ the population density, ‘PM’ the proportion of males in the herd and ‘♂ ASTR’ the male age structure (see text for details). All lines in the diagram represent a specific linear mixed-effects model. The path model in (**b**) shows the standardized coefficients and SEs for paths associated with statistically significant effects. Nonsignificant paths (*P* > 0.05) shown as darker lines in panel (**a**) have been set as light gray lines in panel (**b**); significant paths with good evidence (*P* < 0.05) for an effect as thick solid lines (**b**) and paths with a weak effect (*P* ~ 0.05) as dotted line (**b**)

From previous studies on this population, we had a priori expectations as to which periods of the year and which weather variables are more likely to affect mating time. Earlier calving dates were recorded following warmer temperatures in April-May, as well as lower precipitation and a reduced snow cover in April [36] so early spring appears to be a first key period with influences on reindeer reproduction. Summer weather also play a detrimental role on reindeer and caribou body condition, because warm summer temperatures increase the level of insect activity and therefore insect harassment [25, 35]. Mushrooms are important food sources for reindeer in autumn and in years where they are particularly abundant, females are in better condition during rut [37]. Accordingly, we recently found that a warming trend in early autumn by being detrimental to mushrooms growth and development, affected the reproductive success of females and delayed the calving date the following spring [38]. The weather conditions around the time of mating are another critical period for reindeer breeding success. From those findings, specific hypotheses could be derived for reindeer mating time (‘MT’), including both timing of males’ rutting behaviors and the dates when females are successfully copulated (i.e. date of an observed copulation that led to the birth of a calf the following calving season). (1) Reindeer mating time would be positively affected by temperature and precipitation in late winter/early spring through indirect effects on spring vegetation productivity and on individuals’ regain of fat reserves [24, 39] but negatively by snow cover through direct effect on the energetic costs of individuals of movement on snow [27, 28]. (2) Reindeer mating time would be delayed following warmer summer temperatures through indirect effect on the level of insect harassment and therefore summer foraging conditions [25, 35]. (3) Weather conditions detrimental to mushrooms development (i.e. warmer temperatures) around the time of mating might cause a delay in mating time through an indirect effect on individuals’ physical condition [38]. Although we had clear hypotheses, and to ensure a fully objective evaluation of the potential effects of weather on mating time, we used a sliding window approach to find the critical time window for each weather variable [26], with the time windows varying by the start date and on a weekly basis (as in [40]. Further, we also considered some population variables known to have an influence on reindeer mating time as the changes in those variables (mainly caused by management practices) can potentially reinforce or dampen climatic effects on mating time [41]. The population variables included sex ratio [42–44], density [21, 45] and male age structure [44]. Based on all of these studies, tentative path models on how climatic variability probably affects reindeer mating time can be built (Fig. 1a). To investigate the direct versus indirect effects of weather on mating time, path analysis can be employed [46, 47]. In the present study, we thus aimed to: (1) quantify the rate of change over time of reindeer mating time, (2) determine whether phenological change in mating time was explained by climatic drivers, and which time windows of those climatic drivers best explained variation in mating time and (3) assess the direct and indirect (through individuals’ pre-rut body weight) effects of the climatic drivers identified on mating time.

## Results

### Temporal trend in mating time

**Fig. 2:**
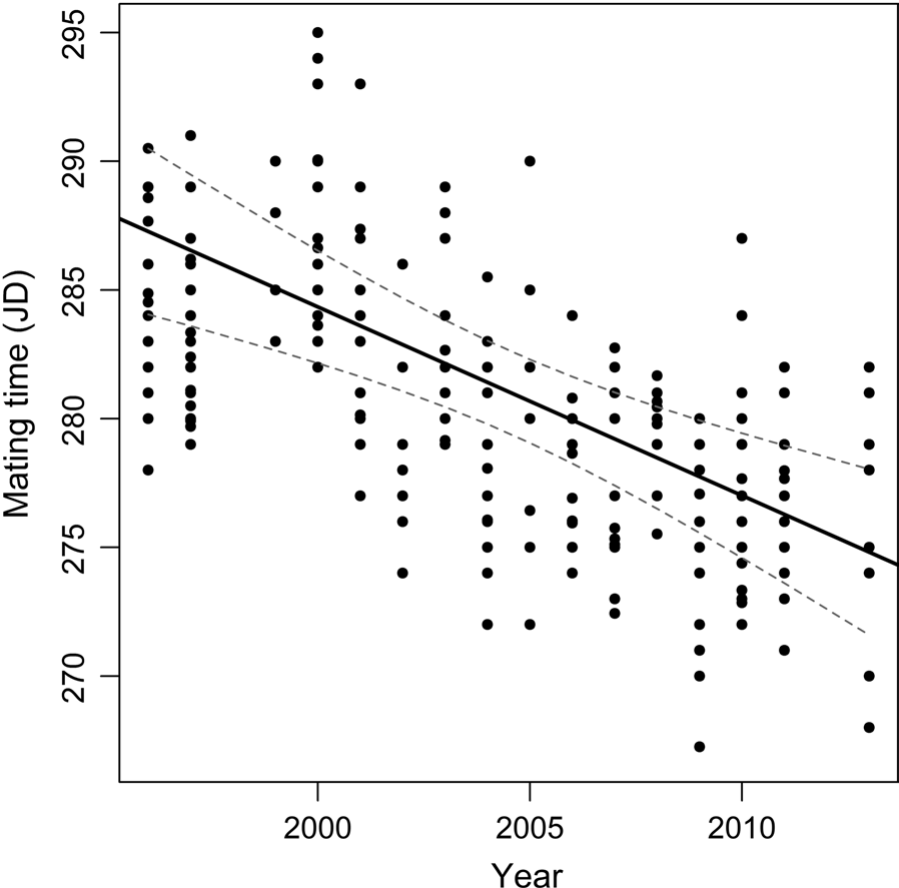
Inter-annual variation of mating time from 1996 to 2013 of a semi-domesticated reindeer population at Kutuharju, northern Finland. Fitted line as well as 95% confidence interval band are provided. The dates are expressed in Julian day (JD) starting January 1st. Data points were weighted by inverse variance (i.e. regression slopes)

From 1996 to 2011 (except years 1998 and 2002), 1,441 males’ mating behaviours were available from 57 different males. From these males, a total of 78 different males’ mating behaviours were available when averaged per male and per year. From 1996 to 2013 (except years 1998, 2008 and 2012), 198 copulation dates were used from 122 different females. Altogether, 276 dates were available from 179 different individuals to estimate the reindeer mating time (‘MT’). The years excluded from the analyses were dropped simply because no data were available those years. The peak date for reindeer mating time is the October 9^th^ and varied significantly between-years (one-way analysis of variance, *F*_(15, 260)_ = 12.62, *P* < 0.001). Between 1996 and 2013, the reindeer MT significantly advanced, by an estimated 0.70 day per year (95% CI [− 1.05; − 0.33]; Fig. 2); leading to an overall shift estimated to about 11 days across 16 years (Fig. 2).

### Critical time window of weather variables

After comparison of the AIC values of the models containing various combinations of weather variables, the critical windows of each weather variable that best explained variation in reindeer MT could be identified. The Table 1 provides details of the 15 models of all combinations of the best windows for each weather variable. The most parsimonious model contained the averaged, minimum temperature for 2 weeks between 12 and 25 July (‘MinTemp’), the total amount of precipitation for 8 weeks between 1 August and 25 September (‘Prec’), and the total accumulated snow cover for 6 weeks between 9 April and 20 May (‘Snow’; Table 1). The second most parsimonious model contained, in addition to the previous weather variables, the averaged, maximum temperature for 2 weeks between 13 and 26 July (‘MaxTemp’; Table 1).

**Table 1:** Comparison of linear models testing the effect of various combinations of weather variables on mating time of a semi-domesticated reindeer population in the Kutuharju field reindeer research station in Kaamanen, northern Finland (69°N, 27°E) from 1996 to 2013

Variables	K	AIC	ΔAIC	AIC_wt_
Mating time				
MinTemp + Prec + Snow	5	1777.05	0.00	0.67
MaxTemp + MinTemp + Prec + Snow	6	1778.48	1.43	0.33
MaxTemp + Prec + Snow	5	1786.65	9.59	0.01
MaxTemp + MinTemp + Snow	5	1788.98	11.93	0.00
MinTemp + Snow	4	1790.24	13.19	0.00
MinTemp + Prec	4	1790.48	13.42	0.00
MaxTemp + MinTemp + Prec	5	1791.48	14.42	0.00
MaxTemp + Snow	4	1796.92	19.87	0.00
MaxTemp + MinTemp	4	1797.38	20.32	0.00
MinTemp	3	1798.73	21.67	0.00
MaxTemp + Prec	4	1802.59	25.54	0.00
MaxTemp	3	1807.86	30.81	0.00
Prec + Snow	4	1813.57	36.51	0.00
Prec	3	1844.42	67.36	0.00
Snow	3	1854.79	77.74	0.00

The linear models had weather variables as fixed effects and year as a random effect. A total of 15 models were fitted. The models were compared and ordered by AIC values. K represents the number of weather variables fitted in the model. The ΔAIC (difference with the AIC of the best model) and AIC weights (AIC_wt_, weight of the model relative to all 15 models fitted) were also provided (see text for details). The dates defining the critical time window for each weather variable are given in *Results*

### Path analyses

The design of the hypothesized path model for reindeer MT is depicted in Fig. 1a. The same path model but keeping only the significant paths (i.e. statistically significant path coefficients) is shown in Fig. 1b. The model was supported as providing a good fit to the observed data, indicated by a non-significant P-value of the goodness-of-fit (χ^2^ = 4.3, df = 6, *P* = 0.64). Starting from the second most parsimonious model (i.e. also including the maximum temperature between 13 and 26 July) led to the same path model, with the same statistically significant paths.

**Fig. 3:**
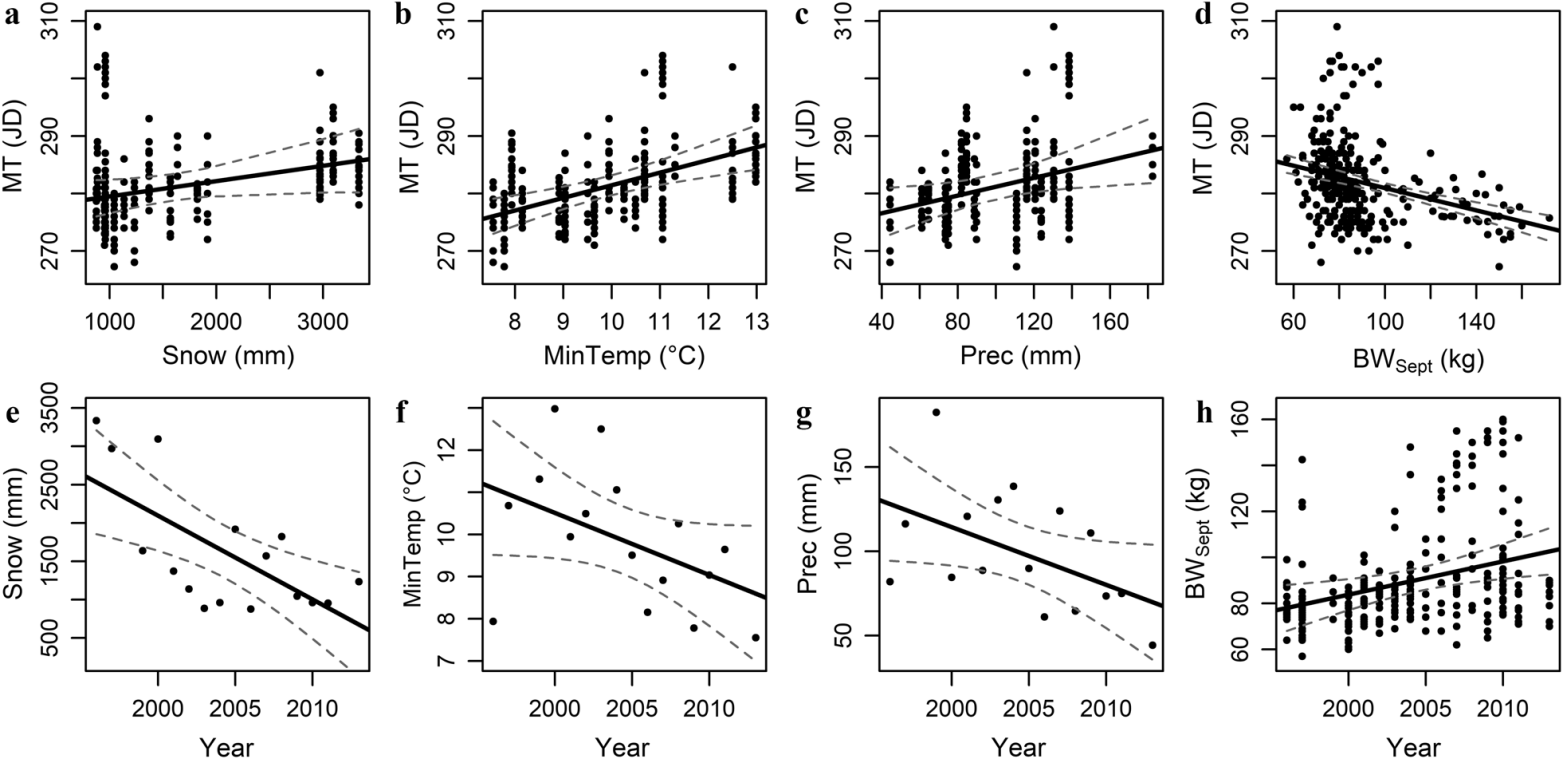
Response of mating time (‘MT’) of a semi-domesticated reindeer population in northern Finland between 1996 and 2013 to (**a**) the total snow cover between 9 April and 20 May (‘Snow’), (**b**) the minimum temperature between 12 and 25 July (‘MinTemp’), (**c**) the amount of precipitation between 1 August and 25 September (‘Prec’), and (**d**) the individuals’ body weight in September (‘BW_Sept_’). The reported temporal trends of those variables were (**e**) a decreasing snow cover in early spring, (**f**) a decreasing minimum temperature in the last 2 weeks of July, (**g**) less precipitation in August–September and (**h**) an increasing pre-rut body weight of individuals. All dates are expressed in Julian day (JD). Graphs are presented with the 95% confidence interval band around the fitted line

From the path model, several results can be drawn (Fig. 1b). The snow cover between 9 April and 20 May, the minimum temperature between 12 and 25 July, the amount of precipitation between 1 August and 25 September, the pre-rut body weight of individuals and the proportion of males in the herd (Fig. 1b) directly affected the mating time. A delay in MT was observed when the snow cover increased from April to mid-May (Snow, *P* < 0.05, Fig. 3a), when the minimum temperature from mid-July to the end of July increased (MinTemp, *P* < 0.05, Fig. 3b) and when the amount of precipitation in August–September was higher (Prec, *P* < 0.05, Fig. 3c). On the other hand, the MT was occurring earlier when the individuals’ body weight in September (BW_Sept_) was higher (*P* = 0.007, Fig. 3d) and when a higher number of males (PM) was present in the herd around the time of the rut (*P* < 0.05, Fig. 1b). The mating time was also indirectly affected by the snow cover between 9 April and 20 May through the direct effect of the snow cover on the individuals’ pre-rut body weight (*P* = 0.005, Fig. 1b). The age of the individuals had a strong, statistically significant positive effect on their body weight in September (*P* = 0.005) but the age did not influence directly the MT (Fig. 1b).

### Temporal trends in weather and population variables

The phenological change of an earlier reindeer MT overtime followed the statistically significant temporal trends of a decreasing snow cover between 9 April and 20 May [*b* = –136.6, 95% CI (− 154.0; − 119.2), Fig. 3e], a colder minimum temperature between 12 and 25 July [*b* = -0.15, 95% CI (− 0.19; − 0.11), Fig. 3f] and less precipitation between 1 August and 25 September [*b* = -2.33, 95% CI (− 3.03; − 1.64), Fig. 3g]. An improvement in the pre-rut body weight of individuals [b = 1.29, 95% CI (0.72; 1.85), Fig. 3h] and more males present in the herd around the time of the rut [*b* = 0.008, 95% CI (0.005; 0.01)] also caused an earlier MT from 1996 to 2013 in this population.

## Discussion

Confirming our hypothesis, the mating time of our reindeer population varied in response to weather variables at different key periods in the annual breeding cycle of reindeer: early spring, summer and end of summer/early autumn. The phenological advancement in reindeer mating time also followed the climatic changes recorded in the study area. Both direct (i.e. thermoregulation) and indirect (i.e. plant growth and food availability) effects of weather conditions may have important influence on herbivore phenology and demography [29]. Therefore, the observed relationships between phenology and weather variables in our study population were interpreted by dissociating the direct and indirect (i.e. through body weight) effect of weather on reindeer mating time.

### Temporal trend of the mating season

The mating season of the semi-domesticated reindeer population of the Kutuharju field reindeer research station in Kaamanen, northern Finland has advanced significantly by 11 days over 16 years (Fig. 2). The reproductive season of *Rangifer* occurs in a highly synchronous, brief period among individuals, with 90% of females impregnated during a period lasting 10–21 days in the end of September or early October in reindeer [16] and 80% of 64 conceptions that occurred the first 11 days of a 4–5 week mating period in caribou [14]. An advancement of 11 days in just 16 years for MT thus represents an important change in the mating season of reindeer even if most of the reindeer mating would remain (to date) in its historical time window. A phenological rate of change of − 7.0 days.decade^−1^ for MT reported in our study population fell in the range of the reported rates of shift in spring phenology of − 9.6 days.decade^−1^ for mammal species [48] and of − 5.1 days.decade^−1^ for temperate-zone species [49]. The breeding phenology of a red deer population was reported to have advanced by between 5 and 12 days across a 28 year study period, with a rate of advancement of 0.26 days.year^−1^ for females’ estrus date and 0.21 days.year^−1^ for males’ rut start date [22]. Similarly, Post and Forchhammer [4] reported that the onset of calving season in a caribou population in West Greenland has advanced by 0.29 days.year^−1^ between 1993 and 2006. Therefore, the rate of phenological change reported in our reindeer population matched with the rates recorded broadly for mammals but was much higher than in other species of the same family. This would suggest that our population is more plastic to environmental change, with a greater ability to track environmental cues and can thus adjust mating time at a faster rate. The underlying explanation would be that supplemental feeding given to the animals in late winter by contributing to improve their body condition might render them physiologically able to be more plastic to environmental change than natural populations [6, 50]. Unfortunately, we were unable to test this assumption with certainty due to the lack of detailed information on the duration or the amount of supplemental feeding given every year to the animals. Alternatively, the abiotic changes in the Arctic exceeding those in temperate, tropical and montane biomes [34, 35], would cause animals’ phenology of reproduction to advance at a faster rate to keep up with their respective changing climate [51]. Either way, it points out the need of proper consideration of site/species specific differences when discussing climate-phenology relationships. Our study can, however, be added to the growing body of literature showing the significant impact of recent climatic changes on the alterations of animal and plant populations’ phenology [49].

### Effect of early spring snow cover on mating time

Snow cover in early spring had a direct and indirect effect on mating time (Fig. 1b), with an earlier mating time (Fig. 3a) following the temporal trend of a decreasing snow cover in April–May (Fig. 3e). Early spring is a key period for reindeer in Arctic since the individuals’ body mass is at its lowest point at that time [30, 52] and they have to recover from winter harshness while availability of food is reduced due to hard and thick snow cover [53]. Availability of food will therefore depend on the emergence of snow free patches allowing reindeer to have access to lichens and dwarf shrubs [54]. Early spring is also the time preceding the calving season and females have already been shown to give birth earlier when an improvement in weather conditions at that time (i.e. warmer temperatures in April–May, lower precipitation and a reduced snow cover in April) was recorded [36]. Recent climatic changes in the Arctic, resulting in warmer temperatures in spring, an earlier timing of snowmelt and changes to hydrologic regimes have in turn advanced the onset and extended the vegetative growing season (review in [55]. Altogether, the ability of females to give birth earlier when the snow cover in April is reduced, combined with an earlier snow melting and reduced energetic costs due to the movement on snow [28] might have helped individuals to start recovering their body weight and replenishing their fat reserves sooner in spring [24, 39]. In turn, an increase in total forage biomass and nutritional content in the Arctic [56] was recorded following such climatic changes. A better spring nutrition, in complementarity with supplemental feeding given in late April and less energetically costly movements on snow [28], could have helped improving the pre-rut body weight of individuals and might explain the lagged effect of an increase of individuals’ body weight in September (Figs. 1b and 3h) after a decreasing snow cover in April–May (Figs. 1b and 3e). An earlier mating season was then recorded (Fig. 3a, d). October body mass in Svalbard reindeer was also shown to increase as a result of greater plant productivity [30]. A delay in mating season due to poor body condition has already been highlighted in other ungulate species (bighorn sheep (*Ovis canadensis*): [8], red deer: [57], elk (*Cervus elaphus*): [58], moose (*Alces alces*): [59]. However, our study appears to be the first to reveal the key role that weather conditions may be having on this pattern.

### Effect of minimum temperature in July on mating time

The mating time was also directly affected by the minimum temperature in the last 2 weeks of July (Fig. 3b), with a decreasing minimum temperature in July overtime (Fig. 3f) inducing an earlier mating season (Fig. 3b). Surprisingly, however, the effect of minimum temperature in the last 2 weeks of July on mating time was not mediated through the individuals’ pre-rut body weight (Fig. 1b). We suspect that the coincidence of a lower level of insect harassment caused by colder minimum temperatures in July (Fig. 3f) might improve reindeer foraging conditions. Beginning in July, forage quality declines as plants mature and fiber accumulates, while insect harassment from primarily skin warble flies *Hypoderma tarandi* (Oestridae) and nasal bot flies *Cephenemyia trompe* (Oestridae) increases. Harassing insects were shown to be detrimental to autumn body weight (carcass weight) of ungulate species [25], by preventing them from feeding effectively [60]. The blood-sucking insects induce in reindeer a behavioral change of a reduced grazing time and an increased energy expenditure caused by their disturbance [25, 60], review in [61]. In addition, several studies have already reported summer grazing conditions to be the main factor affecting growth rate and body size in reindeer/caribou (review in [60]. Although this effect was not strong enough to be observed in our reindeer population, we hypothesize that harassing insects by reducing the ability of individuals to feed optimally during the summer period could still have influenced the mating time, causing a delay when the minimum temperature in July is higher.

### Effect of end of summer/early autumn precipitation on mating time

Another direct effect of a weather variable was observed on mating time without an indirect effect mediated via the pre-rut body weight of individuals: a decreasing amount of precipitation between 1 August and 25 September overtime (Fig. 3g) induced an earlier mating time (Fig. 3c). In a population of red deer, increased precipitation around the time of mating was shown to cause a delay in females’ timing of estrus and thereafter on birth dates [50]. It was hypothesized that increased rainfall at that time of the year could have resulted in environmental deterioration and reduced food availability for females [62] or more directly, that increased rainfall may have impacted the physiological condition of females, for example through increased thermoregulatory costs [50]. Perhaps the same phenomenon is under way in our population, although the direct effect of August–September rainfall on the body weight of individuals in September is not strong enough and so could not be observed. We speculate that a combination of socio-biological factors are involved in this population and thus that a potential indirect effect of weather variables on mating time through the pre-rut body weight of individuals may be hidden. This might be the case if we consider the direct effect that some social factors (e.g. proportion of males in the herd, density of the population, etc.) might have on the body weight of individuals. On the other hand, *Rangifer* species draw on body fat reserves to sustain maintenance costs when weather conditions reduce forage availability and accessibility [63]. The individuals’ fat reserves could therefore partially buffer the effects of weather variables on their pre-rut body weight to still insure a successful reproduction, although the mating time would still rely on environmental cues.

### Limitations

Supplementary winter feeding in semi-domesticated reindeer populations is used as a common management practice to buffer the effects of environmental stochasticity on the body condition by protecting individuals from late winter starvation [64] and has started to become a management practice only since the 1980s in the northern part of Finland [54, 64]. Therefore, whether the improvement in individuals’ body weight in September (Fig. 3h) is attributable to supplemental feeding only or to a combination with a better food availability in the natural environment is impossible to disentangle in this study. Despite supplemental feeding, that occurs regularly in late April, we still found a direct effect of certain weather variables on reindeer mating time. This suggests that animals would still be sensitive to weather conditions as environmental cues to adjust their reproductive phenology. In a recent study, environmental factors were shown to affect Julian birth date and birth mass of white-tailed deer (*Odocoileus virginianus*) even though mothers were continuously allowed access to a high-quality diet [65]. The study on white-tailed deer therefore demonstrated first that environmental factors may have a greater influence on reproductive outputs than previously supposed in ungulates and that constant supplemental feeding was not enough to curtail the environmental effects on reproductive traits. However, the direct effects of weather variables on mating time suggest that other mechanisms (other than just the body weight) might be responsible for the plastic response of reindeer mating time to environmental change. As such, the amount of precipitation between 1 August and 25 September was reported to have a direct effect on reindeer mating time (Figs. 1b and 3c) but without a mediated effect on individuals’ pre-rut body weight (Fig. 1b). The causal effect of weather conditions on seasonal timing of animals is still an unsolved mystery that we have just started to explore. For instance, Caro et al. [51] have proposed that the thermoregulation might be the starting point explaining the link between ambient temperature and seasonal timing of endotherms, through several effector pathways: thyroid hormones, prolactin, melatonin and the preoptic area. Understanding how the body perceives other environmental cues (e.g. precipitation), integrates it into the neuroendocrine system, and translates it into effector mechanisms that shape seasonal timing is still a major challenge [51].

## Conclusions

The timing of mating has occurred earlier in response to a decreasing snow cover in early spring, colder minimum temperatures in the last 2 weeks of July and less precipitation in August-September in a semi-domesticated reindeer population in Finnish Lapland. An improvement in individuals’ pre-rut body weight, certainly mediated by an earlier snowmelt and less energetically costly movements on snow in early spring [66] might have contributed to such observed phenological change. Also, a lower level of insect harassment in July might have improved individuals’ foraging conditions, after which they have mated earlier in autumn. As in a population of red deer [22], less precipitation in August-September induced an earlier timing of mating, without an indirect effect of this weather variable on the pre-rut body weight of individuals.

Despite supplemental feeding in the semi-domesticated populations, reindeer populations may therefore be more responsive to climatic variability than previously acknowledged [61]. Birth dates of a given female did not respond to increasingly earlier onset of spring across years in roe deer [67], and the explanation proposed was that the ovulation and conception dates of roe deer appear to be under the control of photoperiod [68]. On the other hand, reindeer birth dates were occurring earlier following better weather conditions in early spring (i.e. warmer temperatures, lower precipitation and a reduced snow cover, see [36] and red deer’s calving dates were delayed following higher autumn rainfall [50]. That both reindeer and red deer showed a plastic response of calving dates to weather variables suggest that capital breeders as a whole could use photic periodicity, in interaction with weather variables as environmental cues to time seasonal reproduction. The mechanism being invoked is that the plasticity in the allocation of their endogenous provisions towards reproduction would allow animals to adjust their timing so that the peaks in resource availability and energy demands are appropriately synchronized [6]. If animal species are able to reliably follow environmental cues (i.e. other than just photoperiod) to time their reproductive efforts, then their viability and survival should be ensured even in case of unusual climatic variability. As pointed out before, the changes in winter weather, with related effects on winter food availability, along with the changes in vegetation spring green-up and its consequences for summer food availability are certainly key factors in forecasting the future of *Rangifer* in tundra ecosystems [52, 61].

## Methods

### Study area and reindeer population

The data is from the Kutuharju field reindeer research station in Kaamanen, northern Finland (69°N, 27°E). Open birch *Betula spp.* and pine *Pinus sylvestris* forests, bogs and lakes dominate the area and the landscape varies between 185–370 m above the sea level. A semi-domestic reindeer population of about 100 animals per year was used in this study. Reindeer were all of known age and individually recognizable thanks to the long-term book-keeping of the herd demography and by marking all of them by collars and ear tags. Since 1996, males were fitted with VHF radio collars while females were fitted with coloured collars, both with unique identification facilitating the monitoring of individual behaviour. Most of the year, reindeer were free ranging in two large fenced enclosures, the north-west section (Lauluvaara ~ 13.8 km^2^) and the south-east section (Sinioaivi ~ 15 km^2^). Every day during the rut period from mid-September to mid-October the collared males and their harem were located and the group composition and all males’ mating behaviours recorded. All the copulations observed in the field were also recorded. After the mating season in late October, the animals were gathered and taken to a winter area (15 km^2^) where they can graze freely on natural pastures. By the end of winter, females were transferred into a calving enclosure (approximately 0.5 km^2^) where calving dates have been recorded. In late winter and especially after harsh winters, the animals were supplementary fed (pellets and hay). Given the significant between-years variability in both males’ (one-way analysis of variance, *F*_(12, 65)_ = 8.97, *P* < 0.001) and females’ body weight in September (*F*_(14, 183)_ = 4.20, *P* < 0.001), we believe that regular supplemental feeding alone could not buffer climatic effects by keeping up individuals’ body weight at a stable level. Unfortunately, no detailed information was available on the duration or the amount of supplemental feeding given every year to the animals.

### Mating behaviours

Males mating behaviours were observed using the focal observation technique [69]. Priority was given to the dominant males as they perform most of the mating behaviours during the rut period (e.g. chasing other males, grunting, herding females, etc., see [70] for further details). The dominant males in reindeer can be easily identified as ‘harem holders’, i.e. occupying a central position in the group (contrary to the ‘satellites’). One dominant male was observed for 15 min and every 15 s, the activity of that male (rest, feed, stand, and walk) was recorded as well as his mating behaviours. The mating behaviours used in this study included ‘Herd’, ‘Chase females’, ‘Spar’, ‘Fight’, ‘Displace’, ‘Chase’, ‘Flehmen’, ‘Investigate’, ‘Sniff’, ‘Attempt copulation’, ‘Court’, ‘Follow female’ (see [71, 72] for further details and description of the behaviours).

### Mating time

The mating season of ungulates starts when male exhibit all behaviors and activities associated with the rutting season (e.g. holding and defending a harem of females; [22]. In red deer, it has been estimated with roaring dates and sexual aggregation patterns [73] and with estrus dates as a cue for the rut period [22]. For reindeer, the rutting season of dominant males was shown to follow a specific sequence: first herding, then chasing other males – or any other agonistic interaction as competition behaviours exhibited between males, and finally investigating and courting females [72]. Using the mating behaviours that follow this sequence, the males’ mating behaviours were beforehand averaged per year and per male to avoid having data nested across multiple hierarchies. From the observed copulations, we kept only the copulation dates that led to the birth of a calf the following calving season and within the gestation length range of 211–229 days [74] to make sure that females were in estrus those dates. The reindeer MT was thus represented by a combination of both males’ timing of rutting activities (n = 78)—the averaged day of the year when males displayed their mating behaviours – and the day of the year when copulations were observed (n = 198). In addition to the date of the reproductive event, the dataset included the year of study, the individual’s identity together with its body weight in September and age. All calendar dates were converted into Julian days starting on 1 January for analysis purposes. In total, 15 years of data from 1996 to 2013 were available to represent the reindeer mating time.

### Population variables

To control for the effect of proportion of males on mating time [42–44], the proportion of males during the mating season was estimated per enclosure as the number of males divided by the number of females over 1 year of age present in that specific enclosure. Between 1996 and 2013 (except 1998), the herd was subjected to a number of experiments including manipulation of the proportion of males, leading to the simultaneous use of the two large enclosures, Sinioivi and Lauluvaara. Consequently, the proportion of males was estimated per enclosure for those years. Thanks to the book-keeping of the herd, the identity of the animals involved in any experiment was known, as well as their presence in each enclosure and therefore allowed to relate every mating behavior exhibited by a male and every copulation date to the corresponding, estimated proportion of males in that enclosure. The effect of proportion of males on MT was thus accounted for in the analyses. In addition to the proportion of males, we also estimated the population density per enclosure-year as the number total of individuals present in a specific enclosure for a given year in order to account for the effects of population density on MT [21, 45]. Because male age structure (♂ASTR) influence mating time [44], it was another population parameter taken into consideration in our study. During the rutting periods from 1996 through 2011, the composition of the male segment of the Kutuharju reindeer herd was manipulated. Three male age structures categories were used during the mating season: (1) only adult (≥ 3 years old) males present, (2) only young males (1.5 years old) present, and (3) a mixture of male age classes, including both adult and young males, present [70]. The indirect effect of climatic variability on MT was studied through the direct effect of the weather variables on the pre-rut body weight of individuals. Every year, all animals are gathered in corrals just before the rut period (in September) and different measurements are taken, allowing us to have accurate measurements of pre-rut body weights of males and females (‘BW_Sept_’). Given that all factors linked to physical condition in reindeer interact with each other so that older individuals tend to be heavier [12], the BW_Sept_ was also corrected by the age of the individuals in the models.

### Weather data

From the Finnish Meteorological Institute, three weather stations (Utsjoki, Ivalo airport and Nellim) in northern Finland (68°N, 27°E) were used to obtain local weather data (daily recorded values for temperature, precipitation and snow cover) from 1996 to 2013. Specifically, to estimate the local weather at our study site with as much reliability as possible, the weighted mean by the distance from the weather station to our study site was used. The distance between our study site and each of the weather stations was precisely assessed using their respective GPS coordinates and the Great Circle longitude-latitude calculations tool (https://www.cpearson.com/excel/LatLong.aspx). Precipitation can be either rainfall or snowfall depending on the temperature. Temperature daily values included the minimum, maximum and average temperature recorded that day. To better reflect climatic variability and its effects on reindeer mating time, we preferred to use the minimum and maximum temperature values. A total of four weather variables were subsequently used in the analyses: minimum temperature (in °C, ‘MinTemp’), maximum temperature (in °C, ‘MaxTemp’), total precipitation (in mm, ‘Prec’) and snow cover (in mm, ‘Snow’).

### Statistical analyses

#### Temporal trends

Variation in mating time, our response variable, was analysed using Linear Mixed-effects Models (LMMs), by running the lmer-function in the R package lme4 [75], < www.r-project.org >). Year only was entered as a fixed-effect factor (continuous variable) in the models, and individual identity and year as multilevel random effects to control for repeated measures [76, 77]. Unstandardized values of the temporal trends were reported and the parameter estimates were derived using the restricted maximum likelihood estimates as recommended for mixed effect models. Linear Models (LMs) with year entered as a covariate were applied to test the temporal trends of the weather and population variables. The temporal trends were considered statistically significant if 95% confidence intervals (CIs) of the parameter estimates excluded 0.

#### Critical time window of weather variables

To find the key period of the year having the greatest influence in determining reindeer MT, we used a sliding-window approach [40], separately for each weather variable (temperature, precipitation and snow cover). In this approach, the strength of association between mating time and the mean of a particular weather variable (or sum for precipitation and snow cover), calculated across a certain time period (window), is tested. The time windows tested were estimated by varying the start date and duration of the window by weekly intervals so that the minimum interval would be of 1 week, while the longest interval could be of 52 weeks, and the start date could be anytime from Julian day 1 (January 1st) to Julian day 365 (December 31^+^). Then, the strength of association between each window and MT was calculated to identify the critical time window (of each weather variable) having the greatest influence on MT [26]. To do so, linear models were used with one time window at a time and year included as a fixed effect in order to avoid the potential confounding effect of the year on weather-phenology relationships that can result simply because both variables change across years. The Akaike Information Criterion values (AIC) of those linear models were then compared and the critical window from the model with the lowest AIC was statistically supported as being the most informative. Once the best critical time period was identified for each weather variable, we assessed which combination of the four weather variables had the highest statistical support when included in the same model. A total of 15 models were therefore tested for all possible combinations of the four weather variables (minimum and maximum temperature, precipitation and snow cover). Again, the AIC values were used for model comparison, as well as Akaike weights (AIC weights) to compare the relative performance of the tested models [78, 79]. The delta AIC (${\Delta }_{i}$) was calculated to provide a measure of each model (among the 15 models tested) relative to the model with the lowest AIC value, as a way to indicate the relative support of the best model. The best combination given by the model with the lowest AIC value was subsequently used in the path analyses. The AIC values, ΔAIC and AIC weights were obtained from the aictab-function of the AICcmodavg package in R [75], < www.r-project.org >).

#### Path analyses

To test the direct or indirect (i.e. through individuals’ pre-rut body weight) effects of climatic variability on mating time, we used confirmatory path analysis. Because path analysis can test the structural nature of multiple relationships between different variables [47], we could clearly identify both direct and indirect effects of weather on mating time, while regression analyses only test the dependence of response variables on a set of predictor variables. Confirmatory path analysis also allows to consider a framework accounting for correlations between mating time, population variables and individuals’ pre-rut body weight. Because our study design was multilevel, with repeated measurements taken on the same individuals and observations nested in different years, the standard methods of testing path models based on maximum likelihood are too difficult to apply [47]. Confirmatory path analyses, however, allow intercepts and path coefficients to potentially vary between hierarchical levels (e.g. individual and year). The Shipley’s method based on the concept of ‘d-separation’ was used to test the causal implications of the hypothesized path models (directed separation, [46, 80]. A path model (directed acyclic graph) is formed by a combination of a series of hypothesized causal relationships between pairs of variables (path coefficients), typically represented by a ‘box-and-arrow’ diagram as in path analysis. The causal relationships in the acyclic graph imply a series of independence relations between pairs of variables that will be determined by the graph-theoretic notion of d-separation [46, 80]. The concept of d-separation is defined as the necessary and sufficient conditions for two variables in a path model (without feedback loops) to be independent upon conditioning on another set of variables [46]. The d-separation therefore represents a topological condition of a directed graph, not a statistical condition of empirical data but this topological condition is directly translated to a predicted independence of variables within the model (i.e. a description of the statistical patterns of conditional dependence and independence that would be true in the observed data if they were generated by the hypothesized causal relationships) [80]. The causal relationships represented in the causal graph will then be tested by performing a simultaneous test of all independence claims in that causal graph. A ‘basis set’ is built, implying all of the claims of dependence and independence made by the causal graph. The statistic $C=-2\sum \mathrm{ln}({p}_{i})$, calculated on the independence claims of the basis set, follows a chi-square distribution with 2* k* degrees of freedom, where k is the number of independence claims in the basis set and ${p}_{i}$ is the null probability of the independence test associated with the *i*th independence claim generated by the model [46, 47]. The model is supported if the causal relationships hypothesized in the path model are correct, i.e. if a lack of significant (*P* > 0.05) difference between the observed and predicted pattern of independencies in the basis set is reported [47]. In our study, the approach is extended using linear mixed-effects models to obtain the null probability (${p}_{i}$ for each independence claim (known as generalized multilevel path models; [47].

The causal relationships tested in the path model were hypothesized based on the following aspects:

1. The identified critical time windows of weather variables were expected to have indirect effects on reindeer MT, through their respective effects on the pre-rut body weight of individuals.
2. The critical windows of weather variables were also expected to have a direct effect on reindeer mating time.
3. The documented effect of pre-rut body weight of individuals on mating time was inferred from previous studies, for females [12, 31, 32] and males [33].
4. The age of individuals was also pre-supposed to have an effect on the mating time [32, 59] and the known correlation between the body weight of individuals and their age was inferred from previous studies [12].
5. Relationships between proportion of males and male age structure on mating time were also hypothesized from previous studies [42–44].
6. The population density was hypothesized to have a direct effect on mating time through promiscuity between individuals causing a higher level of sexual biostimulation [14] and an indirect effect through its influence on individuals’ body weight [45].

The hypothesized structure of the path model was shown in Fig. 1a. The conditional independence of pairs of variables was tested in linear mixed-effects models (LMMs), with individual identity fitted as a random effect to account that each individual had multiple records. Year was also fitted as a multilevel random effect, to account for stochastic variation between years. Once the appropriate model was identified (i.e. most parsimonious model given the lowest AIC), the same statistical methods were used to test conditional dependence of pairs of variables (i.e. pairs of variables hypothesized to be correlated). The regression coefficients with their standard errors for each path (path coefficients) were reported if dependence associations were found significant. All variables in the path models before calculation of path coefficients were centred and standardized (X̅ = 0, *SD* = 1) to be on a comparable scale. Analyses were performed in R 3.6.0 [81].

## Data Availability

The datasets used and analysed during the current study are available from the corresponding author on reasonable request.
